# Artificial Intelligence-Aided Microfluidic Cell Culture Systems

**DOI:** 10.3390/bios16010016

**Published:** 2025-12-24

**Authors:** Muhammad Sohail Ibrahim, Minseok Kim

**Affiliations:** 1 Interdisciplinary Research Center for Intelligent Secure Systems (IRC-ISS), King Fahd University of Petroleum & Minerals, Dhahran 31261, Saudi Arabia; muhammad.ibrahim@kfupm.edu.sa; 2 On-Sensor AI Semiconductor Systems, Kumoh National Institute of Technology, Gumi-si 39177, Republic of Korea; 3 Department of Mechanical Engineering, Kumoh National Institute of Technology, Gumi-si 39177, Republic of Korea; 4 Department of Aeronautics, Mechanical and Electronic Convergence Engineering, Kumoh National Institute of Technology, Gumi-si 39177, Republic of Korea

**Keywords:** microfluidics, organ-on-a-chip, artificial intelligence, cell culture

## Abstract

Microfluidic cell culture systems and organ-on-a-chip platforms provide powerful tools for modeling physiological processes, disease progression, and drug responses under controlled microenvironmental conditions. These technologies rely on diverse cell culture methodologies, including 2D and 3D culture formats, spheroids, scaffold-based systems, hydrogels, and organoid models, to recapitulate tissue-level functions and generate rich, multiparametric datasets through high-resolution imaging, integrated sensors, and biochemical assays. The heterogeneity and volume of these data introduce substantial challenges in pre-processing, feature extraction, multimodal integration, and biological interpretation. Artificial intelligence (AI), particularly machine learning and deep learning, offers solutions to these analytical bottlenecks by enabling automated phenotyping, predictive modeling, and real-time control of microfluidic environments. Recent advances also highlight the importance of technical frameworks such as dimensionality reduction, explainable feature selection, spectral pre-processing, lightweight on-chip inference models, and privacy-preserving approaches that support robust and deployable AI–microfluidic workflows. AI-enabled microfluidic and organ-on-a-chip systems now span a broad application spectrum, including cancer biology, drug screening, toxicity testing, microbial and environmental monitoring, pathogen detection, angiogenesis studies, nerve-on-a-chip models, and exosome-based diagnostics. These platforms also hold increasing potential for precision medicine, where AI can support individualized therapeutic prediction using patient-derived cells and organoids. As the field moves toward more interpretable and autonomous systems, explainable AI will be essential for ensuring transparency, regulatory acceptance, and biological insight. Recent AI-enabled applications in cancer modeling, drug screening, etc., highlight how deep learning can enable precise detection of phenotypic shifts, classify therapeutic responses with high accuracy, and support closed-loop regulation of microfluidic environments. These studies demonstrate that AI can transform microfluidic systems from static culture platforms into adaptive, data-driven experimental tools capable of enhancing assay reproducibility, accelerating drug discovery, and supporting personalized therapeutic decision-making. This narrative review synthesizes current progress, technical challenges, and future opportunities at the intersection of AI, microfluidic cell culture platforms, and advanced organ-on-a-chip systems, highlighting their emerging role in precision health and next-generation biomedical research.

## 1. Introduction

Microfluidics is a rapidly evolving field that utilizes micrometer-scale channels, where typical channel dimensions range within tens to hundreds of μm, to control and manipulate fluid volumes ranging from 10^−9^ to 10^−18^ L [1]. A standard microfluidic device incorporates microchannels, inlets, outlets, micromixers [2], micropumps, microchanmbers, and microvalves to manage and process trace fluid quantities [3]. These miniaturized systems enable highly integrated operations at the submicron scale [4]. Owing to these advantages, microfluidic platforms, commonly referred to as lab-on-a-chip systems, facilitate precise and automated chemical reactions as well as biological processes [5]. Microfluidics has been among the frontiers of biological research [6,7] due to its significant advantages and applications in sample preparation processes and various cell culture applications [8,9].

Traditional microfluidic devices are primarily used for fluid manipulation in controlling micro-reactions between various analytes. Microfluidic cell culture systems in biomedical settings, often referred as organ-on-a-chip platforms, utilize microfluidics to simulate in vivo environments while providing sophisticated control of parameters available with in vitro experiments. Standard in vitro experiments and research rely on several platforms such as test tubes, culture dishes, or specialized environments maintained outside the living organism. Maintenance of such in vitro environments pose significant challenges, which can be addressed by organ-on-a-chip platforms due to the exceptional control and flexibility provided by the microfluidic platforms [10]. Such organ-on-a-chip systems leverage microchannels and microchambers to handle microliter- to femtoliter-scale sample volumes to ensure reliable prediction of fluid flow patterns under different flow settings [11].

**Figure 1:**
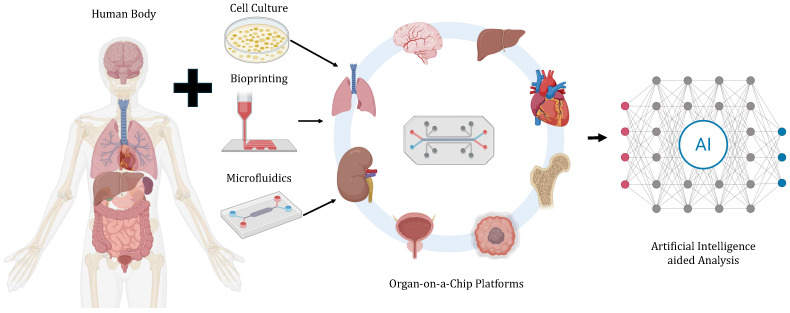
Schematic of AI-aided microfluidic cell culture and organ-on-a-chip platforms. Patient-derived cells and tissues are integrated into microfluidic devices to recapitulate organ-level physiology. Organ-on-a-chip platforms can model systemic interactions, while AI algorithms enable automated data analysis, predictive modeling, and optimization for drug discovery and personalized medicine. Created using BioRender.com.

Microfluidic cell culture platforms can provide significant insights into organ functions and human tissues by enabling the real-time control and monitoring of cells and organs through in vitro research platforms. Such advances are made possible by the integration of various technologies such as the construction of organ tissues and scaffolds by bioprinting, microfluidic control of blood perfusion and sheer stress, and cell culture technology, enabling the control of oxygen gradient and nutrient levels, among others [12]. Artificial intelligence (AI) provides a number of applications and benefits to organ-on-a-chip platforms in terms of improving efficiency and accuracy, providing insights by leveraging AI-assisted imaging analysis, predictive modeling, etc. Figure 1 presents an overview of the synergistic integration of microfluidic cell culture and organ-on-a-chip platforms with AI technology to enhance the efficiency, accuracy, and predictive modeling of the present organ-on-a-chip and microfluidic cell culture systems, paving the way for improved drug discovery, personalized medicine, and healthcare. The integration of AI into organ-on-a-chip platforms enhances the reproducibility of cell culture protocols, thereby reducing variability, strengthening the reliability of conclusions, and advancing in-depth investigations and drug development. By analyzing data from integrated sensors and imaging systems, AI algorithms can identify changes in cell behavior, such as variations in morphology, appearance, or growth rate, and dynamically adjust culture conditions in real time to optimize cell health and growth.

Organ-on-a-chip technology has evolved rapidly since the earliest demonstrations of mechanically active microphysiological systems and organ-mimicking platforms, which established the foundation for modeling human physiology in vitro [13]. Subsequent authoritative reviews have further highlighted the potential of microengineered culture environments to advance drug development, toxicology, and disease modeling [14,15,16]. Parallel progress in AI-assisted microfluidics and lab-on-a-chip automation has been comprehensively outlined in several studies, which discuss machine learning-enabled experiment design, automated imaging analysis, and real-time control strategies [17,18,19]. While these foundational and domain-specific reviews have provided valuable insights, they typically address organ-on-a-chip development or AI methodologies in isolation.

This study aims to comprehensively review recent literature on the integration of AI technologies and algorithms into organ-on-a-chip and microfluidic cell culture platforms. Existing reviews have often focused on specific applications, such as microfluidic cancer models [20], drug screening systems [21,22], toxicology testing [23], or AI-enhanced personalized medicine platforms [24], but do not broadly assess how AI can systematically transform microfluidic and organ-on-a-chip systems across diverse biomedical domains. The present review seeks to bridge this gap by providing a holistic overview that emphasizes the synergy between AI and microphysiological systems, with a particular focus on advancing reproducibility, real-time analysis, closed-loop control, and predictive modeling. Furthermore, we highlight critical challenges in data analysis, domain generalization, model interpretability, and the need for explainable AI approaches, as well as future opportunities for integrating AI-enabled microfluidic platforms into precision medicine workflows.

As AI becomes increasingly integrated into microfluidic and organ-on-a-chip systems, the need for explainable AI approaches has become critical. Many deep learning models used for phenotypic classification or drug-response prediction operate as “black boxes,” making it difficult to understand which cellular or morphological features drive their decisions which is an essential requirement in biomedical and regulatory contexts. For example, in AI-assisted tumor-on-a-chip platforms, convolutional neural networks may distinguish between drug-sensitive and drug-resistant phenotypes with high accuracy, but without explainable AI tools such as Grad-CAM [25] or SHAP [26], researchers cannot determine whether the model relies on biologically meaningful features. Introducing explainable AI into microfluidic workflows will therefore be crucial for ensuring transparency, validating AI predictions against biological mechanisms, and supporting their translation into clinical decision-making. Beyond explainability, several broader limitations remain under active investigation, including challenges in standardizing microfluidic data streams, integrating multimodal sensor outputs, and establishing reproducible AI workflows in dynamic culture environments. At the same time, significant opportunities exist for developing closed-loop AI control, patient-specific disease modeling, and multi-organ platforms capable of supporting precision health applications. These limitations and future opportunities, including the central role of explainable AI, are explored in detail in the subsequent sections of this review.

## 2. Background

### 2.1. Overview of Artificial Intelligence

The origins of AI can be traced back to the 1950s, when researchers first explored the concept of replicating human intelligence in machines [27]. Initial progress in AI primarily revolved around rule-based reasoning and the development of expert systems [28]. However, these early efforts were limited by insufficient data availability, suboptimal algorithms, and limited computational capabilities. The advent of neural network architectures, GPU-accelerated computing, and powerful frameworks such as Keras [29], TensorFlow [30], and PyTorch [31] significantly transformed the field. These advances fueled the rapid expansion of AI, particularly in machine learning and deep learning. While machine learning enables systems to iteratively improve their performance through data-driven learning, deep learning further allows for hierarchical pattern recognition and feature abstraction. Today, AI has become a transformative force across numerous domains, including computer vision, speech recognition, and natural language processing, among others [32,33,34]. The learning paradigms of machine learning, i.e., supervised, unsupervised, semi-supervised, and reinforcement learning, are outlined in Table 1.

AI methods are commonly employed to address four fundamental categories of problems: regression, classification, clustering, and dimensionality reduction. Each category addresses distinct analytical challenges. Regression focuses on predicting continuous numerical outcomes based on input features, such as estimating drug response levels from experimental data [35]. Classification involves assigning data samples into predefined categories, such as distinguishing healthy versus diseased tissue [36]. Clustering seeks to group unlabeled data into meaningful clusters, which is particularly useful in identifying hidden patterns or subpopulations in biological datasets [37]. Dimensionality reduction reduces the complexity of high-dimensional data while preserving essential structures, thereby facilitating visualization and improving computational efficiency [38].

AI techniques, particularly machine learning and deep learning methods, can play a pivotal role in advancing microfluidic cell culture and organ-on-a-chip platforms. These systems generate massive and complex datasets, from imaging, sensor readouts, and continuous monitoring, which are complex for conventional analytical methods. AI techniques enable automated data processing, robust pattern recognition, and predictive modeling of cellular dynamics, thereby minimizing human error and improving reproducibility in culture protocols. In microfluidic cell culture, machine learning algorithms can optimize fluidic parameters, nutrient delivery, and mechanical cues to sustain physiologically relevant environments, while deep learning models can extract high-dimensional features from live-cell imaging to monitor morphology, proliferation, and cell classification in real time. Furthermore, AI models can enhance drug screening and toxicity testing by identifying phenotypic responses and predicting therapeutic efficacy across patient-derived samples.

### 2.2. Microfluidic Technology

Microfluidic technology, often referred to as “lab-on-a-chip,” enables the manipulation of ultra-low fluid volumes in micron-scale channels [1]. These chips integrate functions such as sampling, mixing, separation, detection, and reaction, offering advantages over traditional methods including reduced reagent and sample use, low cost, high throughput, and rapid analysis [39]. A defining feature of microfluidics is their low Reynolds number (Re), which reflects the ratio of inertial to viscous forces. In microchannels, Re is typically low, leading to laminar flow where fluids can move side-by-side with minimal mixing or turbulence [40]. This property underpins diverse applications such as droplet generation, chemical gradient formation, cell migration studies, tissue engineering, and controlled drug delivery [41,42].

Organ-on-a-chip technologies leverage microfluidics to reproduce physiologically relevant microenvironments by simulating fluid shear stress (FSS), mechanical forces, and concentration gradients. FSS in microchannels can be tuned to mimic in vivo conditions that regulate cell morphology, adhesion, proliferation, and differentiation [43]. This parameter has been shown to influence intestinal epithelial behavior [44], vascular endothelial injury [45], tumor cell metastasis [46], and waste discharge in dynamic cultures [47]. Additionally, elastic microfluidic chips can model mechanical stresses such as pulmonary expansion [48], cardiomyocyte contraction [49], and intestinal peristalsis [50]. Microfluidics also enables the maintenance of stable chemical gradients that regulate cell proliferation, migration, and drug responses [51], as well as other microenvironmental factors including ECM stiffness [52] and oxygen concentration [53].

The versatility of microfluidic chips is further enhanced by integration with micropumps, microvalves, microreactors, and advanced manipulation technologies. These platforms can be coupled with detection modalities, including immunoassays [54], fluorescence real-time imaging [55], etc., allowing manipulation from bulk fluids down to single-cell resolution. This precision enables programmed interactions between cells and biomolecules, facilitating applications in cell culturing, organ-on-a-chip systems, disease modeling, and drug testing [56,57,58].

### 2.3. Cell Culture Techniques

The availability of appropriate cell sources remains a critical bottleneck for organ-on-a-chip technologies. Mature somatic cells are typically obtained from hospital patients through surgical procedures, which significantly limits supply. In contrast, stem cells offer a renewable alternative due to their capacity for self-renewal, proliferation, and differentiation into diverse somatic cell types, thereby alleviating shortages of primary cells for chip-based systems [59]. Beyond their role in regenerative medicine, stem cells have become indispensable in organ-on-a-chip research, where they support the creation of physiologically relevant models. Cell cultures also provide a controlled platform to investigate fundamental mechanisms of cellular, tissue, and organ function, as well as disease pathogenesis, thereby informing drug discovery and disease modeling.

Traditional 2D cell culture remains widely used due to its maturity, simplicity, and low cost. In this approach, cells are typically cultured as monolayers on rigid polystyrene-coated plates, which support adhesion and growth [60]. However, compared to the in vivo environment, 2D culture produces cells with altered morphology, limited cell–cell interactions, and reduced physiological relevance. Such discrepancies can distort drug sensitivity and compromise therapeutic predictions [61]. To address these limitations, 3D cell culture systems have been developed to better replicate the in vivo microenvironment.

Three-dimensional cultures can be broadly categorized as scaffold-free or scaffold-based. Scaffold-free systems rely on spatial or mechanical constraints to enhance cell–cell contact, with common approaches including hanging drops [62], magnetic levitation [63], and ultra-low attachment plates [64]. While relatively simple, these methods often yield spheroids of inconsistent size, variable viability, and scalability challenges that hinder clinical translation. Scaffold-based cultures, in contrast, provide physical and biochemical support for cell–matrix interactions. Examples include hydrogels, polymeric scaffolds, and hydrophilic or fiberglass scaffolds [65,66,67]. Hydrogels are particularly advantageous due to their high biocompatibility and ability to mimic ECM properties, creating near-physiological conditions that enhance cell growth and differentiation [68]. Hydrogel scaffolds have been successfully applied for culturing stem cells into tissues such as cartilage, bone, liver, heart, skin, and brain [69,70,71,72,73].

Despite these advances, 3D cultures also present limitations. They often lack the dynamic flow conditions present in vivo, leading to issues with oxygen and nutrient supply as well as potential necrosis within constructs. Additional challenges include inefficient waste removal, difficulties in establishing tissue–tissue interfaces, CO_2_ regulation, and restricted imaging of complex 3D structures. These shortcomings have motivated the development of hybrid approaches that combine microfluidic technology with 3D cell culture, offering dynamic control of fluid flow, nutrient delivery, and mechanical forces to more accurately model physiological conditions.

## 3. Applications of AI in Organ-on-a-Chip Systems

The convergence of AI and microfluidic cell culture technologies has opened new frontiers in biomedical research and translational applications. Microfluidics enables precise spatiotemporal control of cell microenvironments, facilitating reproducible and physiologically relevant culture systems, while AI provides the computational power to analyze complex, high-dimensional datasets generated from these platforms. Together, these technologies are being applied across a wide spectrum of fields, including drug delivery, drug development, and diagnostics; cancer research, ranging from tumor cell classification, monitoring glioblastoma invasion, and mimicking tumor microenvironments to early diagnosis of cervical, colorectal, breast, and nasopharyngeal cancers; stem cell culture and prediction of cell states; and automated species detection in in vivo live-cell imaging. Additional applications include osteoporosis drug testing using bone-on-a-chip platforms, angiogenesis and chronic kidney disease analysis, as well as cardiac and nerve-on-chip studies. By integrating AI-driven image analysis, predictive modeling, and phenotypic profiling, AI-aided microfluidic systems are accelerating breakthroughs in disease modeling, therapeutic screening, tissue engineering, and organ-on-a-chip development.

### 3.1. Applications in Cancer Research

**Figure 2:**
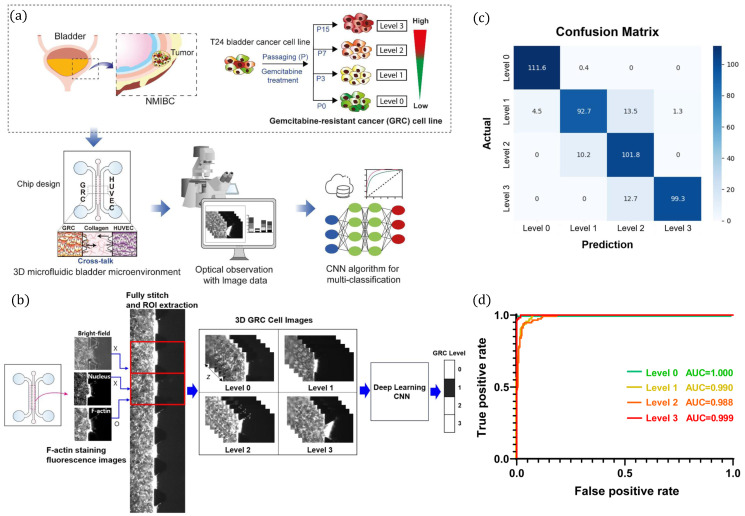
Schematic and performance analysis for predicting anticancer drug resistance using a 3D microfluidic bladder cancer model with deep learning. (**a**) Generation of gemcitabine-resistant T24 bladder cancer cell lines and integration into a 3D microfluidic microenvironment. Images acquired from the chip were analyzed using a CNN for multi-class resistance classification. (**b**) Imaging and analysis workflow, including F-actin staining, bright-field and nuclear imaging, ROI extraction, and reconstruction of 3D GRC images across resistance levels (0–3). (**c**) Confusion matrix demonstrating accurate multi-class classification and (**d**) ROC curves showing high AUC values for all resistance levels (0: 1.000, 1: 0.990, 2: 0.988, 3: 0.999). Reprinted with permission from Tak, Han, Leem, Lee, Paek and Kim. (2024) [74], published by Frontiers under a CC-BY 4.0 license.

Cancer research has increasingly turned to microfluidic cell culture and organ-on-a-chip technologies combined with AI to model tumor microenvironments and predict therapeutic outcomes with higher fidelity. The study in [74] exemplifies this approach by integrating a microfluidic bladder cancer (BC) model with deep learning to predict anticancer drug resistance as presented in Figure 2. The platform cultured the T24 BC cell line across four gemcitabine resistance levels, i.e., parental, early, intermediate, and late, within a collagen-based matrix as shown in Figure 2a. To mimic the tumor microenvironment, these GRC cells were co-cultured with endothelial cells (HUVECs) in adjacent compartments of the microfluidic chip, enabling dynamic cross-talk. High-content imaging generated thousands of bright-field and fluorescence images that captured morphological changes associated with drug resistance. A convolutional neural network (CNN) was then developed, consisting of three convolutional layers, max-pooling operations, and fully connected layers, as shown in Figure 2b, to classify resistance phenotypes. With a dataset of 2674 images augmented for robust training, the model achieved a classification accuracy of 95.2%, with sensitivity and specificity values exceeding 82.8% and 92.2%, respectively, and AUC values above 0.988 (Figure 2c,d). Collectively, this AI-driven microfluidic platform demonstrates how automated image analysis of 3D cultures can capture subtle morphological phenotypes linked to drug resistance, offering a label-free and clinically translatable tool. The study further suggests that the incorporation of patient-derived cells into such systems could provide rapid, individualized prediction of therapeutic responses, advancing precision oncology.

A CNN-based framework, presented in [75], was developed to predict key parameters governing glioblastoma (GBM) cell behavior from fluorescence images in microfluidic cultures. Trained on synthetic data generated from a validated mathematical model. The mathematical model used to generate the synthetic training data is based on a system of coupled reaction–diffusion partial differential equations that describe glioblastoma cell proliferation, migration, and hypoxia-driven phenotypic switching within confined microfluidic geometries [76]. These partial differential equations capture how local oxygen concentration, nutrient diffusion, and cell density regulate the balance between migratory and proliferative behaviors, following well-established advection–diffusion–reaction modeling frameworks commonly used for tumor growth. By incorporating spatial gradients and dynamic feedback between cell populations and their microenvironment, the model reproduces hallmark glioblastoma phenomena such as pseudopalisade formation, necrotic core development, and invasion fronts, providing a biologically realistic basis for training and validating the deep learning approach. The network achieved high accuracy (Pearson’s correlation coefficient ρ > 0.99) in predicting proliferation–migration dynamics under hypoxic conditions. Application to real GBM microfluidic experiments, including necrotic core and pseudopalisade formation, demonstrated strong agreement with experimental results. A hydrogel-based microfluidic device developed to culture U87 GBM cells in a 3D matrix and perform multiplexed drug screening is presented in [77]. The system integrates a membrane-capped 2D array of culture chambers perfused via a microfluidic concentration gradient generator, enabling fractional serial dilutions (1, $\frac{1}{2}$, $\frac{1}{4}$, 0). PEG-based hydrogels supported high cell viability (>90% for 4 days), and diffusion of chemotherapeutics (temozolomide, carmustine) from perfusion channels produced reliable dose–response curves. To quantitatively assess drug efficacy, linear regression analysis was applied to normalized viability data across concentration gradients, resulting in high coefficient of determination ($R^{2}$ > 0.9), confirming the accuracy and reproducibility of the platform. Physically Guided Neural Networks with Internal Variables (PGNNIV) has recently been applied to microfluidic models of GBM to better capture tumor invasion dynamics [78]. By integrating data from microfluidic devices with nonlinear advection–diffusion–reaction equations, PGNNIV can unravel oxygen-dependent “go or grow” metabolic switching in GBM cells. Unlike purely parametric models, this approach provides both predictive and explanatory capacity, enabling accurate simulation of tumor evolution under varying oxygenation conditions. Such integration of AI with microfluidic cell culture platforms not only improves mechanistic understanding of GBM progression but also opens avenues for virtual therapy testing and in silico personalized medicine. A 3D microfluidic GBM tumor-on-chip model, presented in [79], was developed to mimic the perivascular niche (PVN) by integrating patient-derived glioma stem cells (GSCs), astrocytes, and endothelial cells. Triculture conditions enhanced GSC invasion and maintained stem-like phenotypes. Single-cell RNA sequencing, combined with PCA and shared nearest neighbor clustering, identified 15 ligand–receptor pairs, including novel candidates (LGR6, FPR1) driving chemotactic GSC migration toward the PVN. This platform enables mechanistic studies of GSC–stromal–vascular interactions and therapeutic discovery.

**Figure 3:**
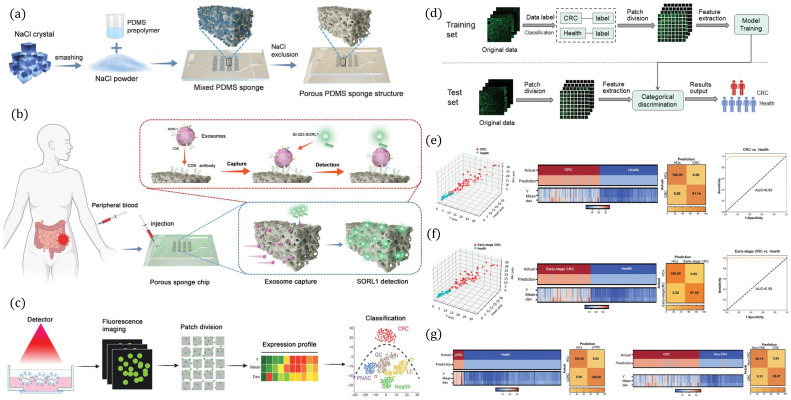
Construction and application of a 3D porous microfluidic chip for exosome SORL1 detection in early colorectal cancer (CRC). (**a**) Fabrication of the porous PDMS sponge chip using NaCl templating. (**b**) Workflow for peripheral blood-derived exosome capture and SORL1 detection. (**c**) Fluorescence imaging, patch division, and expression profiling for CRC vs. healthy classification. (**d**) Machine learning pipeline for feature extraction and classification. (**e**–**g**) Classification outputs, including 3D scatter plots, heatmaps, confusion matrices, and ROC curves for CRC vs. healthy, early-stage CRC vs. healthy, and advanced CRC vs. non-CRC, demonstrating high diagnostic performance (AUC = 0.99). Reprinted with permission from [80], Copyright © 2023 Wiley-VCH GmbH.

The study in [80] presented a porous sponge-based microfluidic platform for exosome detection aided by machine learning-based classification as presented in Figure 3. The fabrication process of a porous PDMS sponge structure using NaCl particle leaching is shown in Figure 3a. NaCl crystals are mixed with PDMS prepolymer, cured, and then removed to generate an interconnected porous network suitable for biomolecule capture. Figure 3b presents the application of the porous sponge chip for liquid biopsy by introducing peripheral blood into the device, enabling exosome capture via surface-immobilized antibodies and subsequent detection of target biomarkers (e.g., SORL1). The chip facilitates sensitive and spatially resolved detection of disease-specific exosomes. Figure 3c presents machine learning-assisted analysis workflow where fluorescence imaging of captured exosomes is performed, followed by patch division and feature extraction to generate expression profiles. Figure 3e–g show that classification using dimensionality reduction and clustering enables discrimination between healthy controls, CRC, early-stage CRC, and other disease subtypes. Performance metrics, including confusion matrices and AUC values, demonstrate high sensitivity and specificity of the platform for disease detection, as shown in Figure 3e–g. A brief summary of AI-aided microfluidic cell culture and organ-on-a-chip platforms for cancer research is presented in Table 2. The table lists various cancers, microfluidic device types, cultured cell or organs, AI algorithms used for analysis, and the performance of such platforms in terms of detection accuracy, AUC, limit of detection (LOD), sensitivity, determination coefficient (R^2^), etc.

### 3.2. Applications in Drug Discovery, Testing, and Screening

Organ-on-a-chip and microfluidic cell culture platforms have been used in various applications related to drug discovery, screening, and analysis [94,95]. However, only a few studies utilize AI methods in combination with the microfluidic cell culture and organ-on-a-chip platforms for applications in drug analysis. In this context, the study in [96] presented an integrated microfluidic platform for precise regulation of cell density using real-time automatic feedback. Human gastric cancer cells were cultured on a PDMS-based chip embedded with interdigital electrodes for impedance-based monitoring of cell proliferation. The impedance signal served as a feedback parameter in a closed-loop system, where a least squares support vector machine (LS-SVM) controller dynamically adjusted flow rates to maintain consistent cell density across repeated assays. The system achieved a low standard error (∼2–3%), ensuring reproducibility and reliability. Such integration of microfluidics with AI-driven feedback control enhances automation in cell culture and provides a robust foundation for high-precision drug screening and delivery studies.

**Figure 4:**
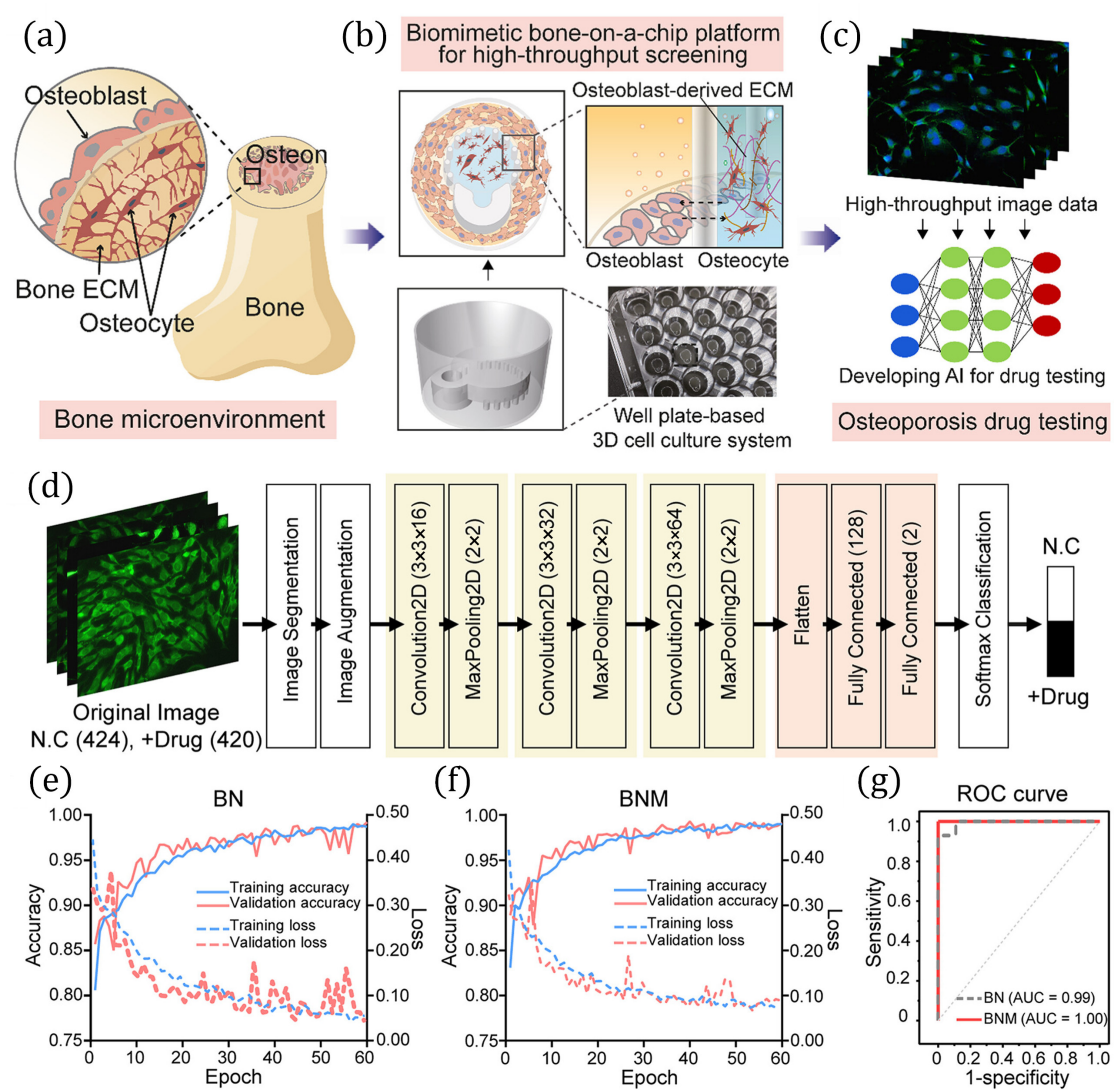
High-throughput biomimetic bone-on-a-chip platform with AI-assisted image analysis for osteoporosis drug testing. (**a**) Schematic of the bone microenvironment containing osteoblasts, osteocytes, and ECM. (**b**) Design of the bone-on-a-chip system using osteoblast-derived ECM in a 3D well plate format. (**c**) Workflow for high-throughput image acquisition and AI-based drug response prediction. (**d**) Deep learning pipeline with segmentation, augmentation, convolution, pooling, and softmax classification for control vs. drug-treated groups. (**e**,**f**) Training and validation curves for BN and BNM models. (**g**) ROC curves showing high classification performance (AUC = 0.99 for BN; 1.00 for BNM). Reprinted with permission from [97], Copyright © 2022 Paek et al. Bioengineering & Translational Medicine published by Wiley Periodicals LLC on behalf of the American Institute of Chemical Engineers.

Figure 4 illustrates a high-throughput biomimetic bone-on-a-chip platform integrated with AI-assisted analysis for osteoporosis drug testing presented in [97]. The system replicates the native bone microenvironment, incorporating osteoblasts, osteocytes, and extracellular matrix (ECM) as presented in Figure 4a. To achieve physiologically relevant culture, osteoblast-derived ECM was embedded in a 3D well plate-based bone-on-a-chip system illustrated in Figure 4b. High-throughput imaging of cultured cells generated large datasets, as presented in Figure 4c, that were subsequently processed using AI-based workflows to evaluate drug responses. The deep learning pipeline, as shown in Figure 4d, consists of image segmentation, augmentation, convolution and pooling layers, and softmax classification to differentiate control and drug-treated groups. Figure 4e,f present training and validation curves demonstrating robust classification performance for β-catenin and nuclear (BN) as well as β-catenin and nuclear and merged (BNM) models, while the ROC curve presented in Figure 4g confirms excellent predictive accuracy with AUC values of 0.99 and 1.00, respectively. A multicellular coculture array microfluidic platform, developed in [98], models liver–immune–skin interactions for predicting adverse cutaneous drug reactions. The system integrates hepatocyte spheroids, keratinocytes, fibroblasts, and immune cells within interconnected compartments, enabling metabolite-mediated cross-talk. Multiparametric cellular readouts were analyzed using SVM models, achieving high predictive accuracy of 87.5% and sensitivity of 100%, signifying superior drug screening and classification capabilities of the microfluidic coculture platform. The study presented in [99] applied a machine learning framework (SperoPredictor V1.2) to repurpose drugs for tubulointerstitial fibrosis (TIF) and identified lubiprostone as a lead candidate. Its antifibrotic effects were validated using a proximal tubule-on-a-chip platform and an in vivo unilateral ureteral obstruction mouse model. The microfluidic organ-on-a-chip provided a physiologically relevant environment for studying fibrosis and drug response, outperforming conventional 2D culture. The work demonstrates how AI-driven drug repurposing integrated with microfluidic models can accelerate therapeutic discovery for kidney disease. A deep learning–based phenotypic drug discovery pipeline (CPHNet) for high-altitude pulmonary edema (HAPE) by leveraging morphological profiling from cell painting images is presented in [100]. Using A549 and HPMEC cells cultured under normoxic and hypoxic conditions, a custom segmentation network (SegNet) was developed for accurate detection of subcellular structures, while a hypoxia scoring network (HypoNet) assessed cellular hypoxia status with a high precision of 0.967. The pipeline enabled large-scale screening of compounds, leading to the identification of two natural products, ferulic acid and resveratrol, with validated anti-HAPE efficacy in both a 3D alveolus-on-a-chip model and in vivo mouse experiments resulting in an average accuracy of 86.8%. By combining cell painting, deep learning, and organ-on-a-chip validation, this work highlights a promising AI-enabled strategy for accelerating therapeutic drug discovery and drug screening in hypoxia-related pulmonary disorders.

**Figure 5:**
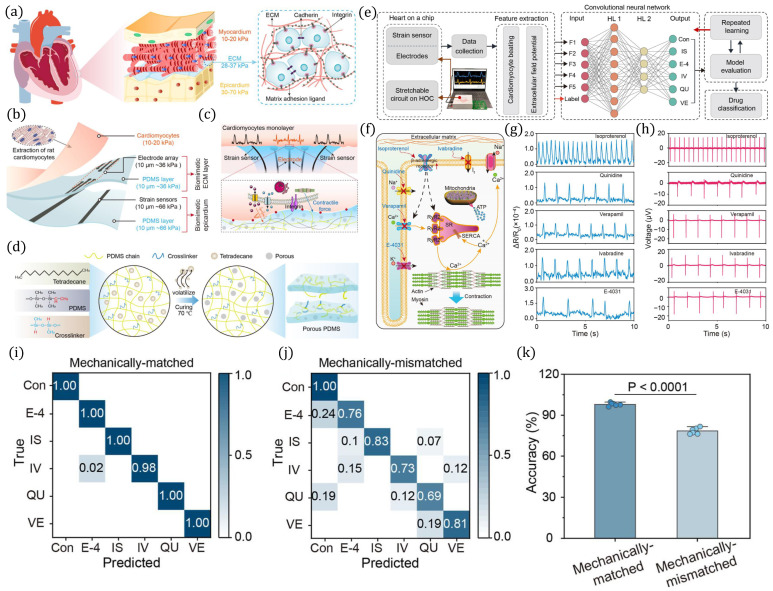
Mechanically matched heart-on-a-chip platform for excitation–contraction coupling analysis. (**a**) Schematic of myocardial mechanics and cell–matrix interactions. (**b**) Isolation of rat cardiomyocytes and integration into a stretchable chip with electrode arrays and strain sensors. (**c**) Cardiomyocyte monolayer used for functional readouts. (**d**) Porous PDMS fabrication for mechanical tuning. (**e**) Machine learning pipeline for drug classification. (**f**,**g**) Representative calcium transients for multiple cardiotropic drugs. (**h**) Extracellular potential recordings under drug treatment. (**i**,**j**) Confusion matrices for drug classification in matched vs. mismatched substrates. (**k**) Comparison of classification accuracy, showing superior performance with mechanically matched systems (*p* < 0.0001). Reprinted with permission from [101], Copyright © 2025 American Chemical Society.

A mechanically matched heart-on-a-chip platform was developed in [101] to better replicate myocardial excitation–contraction coupling and enable AI-assisted drug screening. The system models the mechanical properties of native myocardial tissue and cell–matrix interactions as shown in Figure 5a. Rat cardiomyocytes were extracted and integrated into a stretchable PDMS chip containing electrode arrays and strain sensors for functional measurements, as illustrated in Figure 5b. The cardiomyocyte monolayer, depicted in Figure 5c, cultured on the chip provided high-fidelity readouts of contractile force and electrophysiological activity. To tune the substrate mechanics, porous PDMS was fabricated using a crosslinker–tetradecane strategy, as shown in Figure 5d. The platform was coupled with a machine learning pipeline, as shown in Figure 5e, that processed electrophysiological and mechanical data for drug classification. Functional assays, illustrated in Figure 5f–h, showed distinct calcium transient and extracellular potential responses to different cardiotropic drugs, including isoproterenol, quinidine, verapamil, ivabradine, and E-4031. Drug classification performance was quantified using confusion matrices, as shown in Figure 5i,j, revealing superior accuracy in the mechanically matched system compared to the mismatched one. Overall, mechanically tuned substrates significantly enhanced classification accuracy, as shown in Figure 5k, demonstrating the importance of biomechanical matching for reliable organ-on-a-chip drug testing.

AI-enabled microfluidic and organ-on-a-chip platforms hold substantial promise for advancing precision health by enabling patient-specific modeling and individualized therapeutic prediction. By integrating patient-derived cells or organoids with AI-driven image analysis and phenotypic profiling, these systems can rapidly identify cellular responses to drugs, stratify responders versus non-responders, and uncover subtle morphological or molecular signatures predictive of treatment outcomes [74,93]. Machine learning frameworks capable of analyzing high-content imaging and multiparametric sensor data further enable the construction of predictive models that can support personalized drug selection and dose optimization [97,99]. Moreover, AI-assisted exosome analysis and biomarker detection on microfluidic chips offer minimally invasive avenues for early disease diagnosis and patient monitoring [80]. As these approaches mature, the combination of organ-on-chip platforms with explainable and data-efficient AI will be critical for translating in vitro patient-specific measurements into clinically actionable insights, thereby strengthening the role of microfluidic technologies in precision medicine.

### 3.3. Applications in Diverse Domains

The integration of microfluidic cell culture and organ-on-a-chip systems and AI technologies has expanded their applicability across diverse domains, ranging from the design of biointerfaces and in vivo live-cell imaging to species detection and physiological flow analysis. These approaches further enable real-time culture monitoring, cell cultivation system assessment, and high-speed 3D imaging of complex models such as organoid cultures, offering powerful tools for both fundamental research and translational applications.

**Figure 6:**
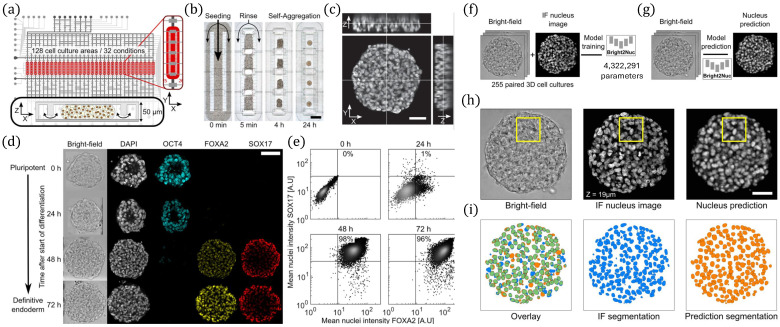
Microfluidic platform for label-free imaging and analysis of 3D pluripotent stem cell differentiation. (**a**) Microfluidic chip design with 128 culture chambers for parallel experiments. (**b**) Workflow for cell seeding, rinsing, and self-aggregation. (**c**) Three-dimensional confocal reconstruction of an on-chip spheroid. (**d**) Time-course immunofluorescence imaging of pluripotency and endoderm markers (OCT4, FOXA2, SOX17). (**e**) Flow cytometry confirming temporal marker expression changes. (**f**,**g**) Bright2Nuc deep learning model development and application for label-free nuclear prediction. (**h**) Comparison of bright-field, immunofluorescence, and predicted nuclear images. (**i**) Segmentation overlays showing high agreement between immunofluorescence-based and Bright2Nuc-predicted nuclei. Reprinted with permission from [102], Copyright © 2023 Atwell et al.

The study presented in [102] introduced a high-throughput microfluidic platform for parallelized 3D stem cell culture and differentiation, integrating label-free imaging with AI-enabled analysis as presented in Figure 6. The device accommodates 128 culture chambers, depicted in Figure 6a–c, allowing automated seeding, rinsing, and self-aggregation of pluripotent stem cells, which subsequently form 3D spheroids, respectively. Differentiation dynamics were monitored through immunofluorescence staining of key pluripotency and endodermal markers (OCT4, FOXA2, SOX17), confirming progressive lineage commitment as shown in Figure 6d. Flow cytometry, presented in Figure 6e, provided complementary validation of temporal expression changes. To overcome the limitations of labor-intensive fluorescence imaging, the authors developed Bright2Nuc, a UNET-based deep learning framework trained on paired bright-field and immunofluorescence datasets to enable label-free nuclear prediction, as shown in Figure 6f,g. This approach achieved high-fidelity single-cell resolution, as demonstrated by strong concordance between predicted and experimental nuclear images depicted in Figure 6h,i. The combination of microfluidics and AI-based imaging thus provides a powerful platform for scalable, dynamic phenotyping of 3D stem cell cultures. A bidirectional adaptive neural interface integrating microfluidics, nanoelectronics, and machine learning was developed in [103] to address key challenges in biointerface design, namely signal stability and power efficiency. The system employed a microelectrode array coupled with a microfluidic chip hosting spatially ordered neuronal cultures, alongside an artificial neural network model trained to classify spatiotemporal neural activity patterns. Hardware implementation using memristor-based synapses demonstrated reliable performance despite device variability, while electrode-shifting strategies prolonged interface functionality. Overall, this work highlights a scalable test-bed for ex vivo studies and establishes a technological foundation toward self-adjusting, low-power biointerfaces for future bioprosthetic applications. The work in [104] introduced artificial intelligence velocimetry (AIV), a physics-informed neural network framework that integrates imaging data with fluid dynamics to infer 3D blood-flow velocity, pressure, and stress fields from 2D images. Using a microaneurysm-on-a-chip platform mimicking diabetic retinopathy, AIV outperforms conventional methods by accurately estimating hemodynamic metrics such as wall shear stress without requiring boundary conditions. The approach enables physiologically relevant analysis of microvascular flow, offering a powerful tool for studying vascular pathophysiology and advancing in vitro and in vivo diagnostics. A microfluidic imaging platform coupled with machine learning for species-level detection in mixed cyanobacterial biofilms from cold environments is presented in [105]. Hyperspectral CNN models achieved superior classification, with mean F1 scores of 0.806 (spectral), 0.887 (spatial), 0.910 (spectral–spatial), and 0.914 (true hyperspectral). The approach outperformed conventional ratiometric methods, enabling robust analysis of morphologically similar filamentous species and offering a scalable tool to study biofilm dynamics and environmental adaptation in polar ecosystems. Table 3 summarizes recent applications of AI-integrated microfluidic platforms across diverse biological systems. The table highlights the types of microfluidic devices, target cells or organ models, applied AI algorithms, and reported performance metrics, demonstrating their broad utility in areas ranging from microbial cell culture monitoring and angiogenesis analysis to pathogen detection, tissue modeling, and neurological research.

## 4. Discussion

Microfluidic cell culture systems and organ-on-a-chip platforms are increasingly generating large volumes of complex, multidimensional data from high-resolution imaging, time-lapse monitoring, and multiparametric readouts. These datasets strongly benefit from the advanced data processing, feature extraction, and predictive capabilities of AI, particularly machine learning and deep learning models. However, current studies primarily employ AI as a supplementary analysis tool rather than embedding it into fully designed experimental pipelines. The development of AI-integrated frameworks, where data acquisition, processing, and interpretation are linked through closed-loop feedback, could significantly enhance experimental design and system performance. Such intelligent pipelines would allow adaptive control over culture conditions and organ models, ultimately enabling more physiologically relevant and reproducible outcomes. The integration of explainable AI is particularly crucial to maintain interpretability and trust in biomedical applications, where the lack of explainability of many models currently limits widespread adoption.

A major insight emerging from the applications presented in Section 3 is that the integration of AI is increasingly transitioning from post-processing tools towards their role as a functional component of microfluidic and organ-on-a-chip systems. In cancer models, for example, deep learning enables high-resolution phenotyping of drug-resistant or invasive cell states, allowing microfluidic platforms to detect subtle morphological transitions that are difficult to quantify manually [74]. Similarly, AI-assisted drug screening systems leverage real-time sensor data, impedance measurements, and multimodal imaging to adjust flow rates, maintain reproducibility, and classify drug responses with high sensitivity [96,97]. Beyond the cancer and drug screening examples discussed in earlier sections, AI-enabled microfluidic cell culture technologies are increasingly being applied to diverse biological systems such as microbial cultures, pathogen detection, angiogenesis models, and nerve-on-a-chip platforms. Table 3 summarizes these emerging areas and highlights the growing versatility of AI–microfluidic integration, underscoring how the field is expanding beyond traditional biomedical applications. These studies collectively demonstrate how AI enhances the predictive power, throughput, and automation of microphysiological systems. More importantly, they reveal a shift toward closed-loop AI control, where models trained on experimental data dynamically regulate culture conditions and improve assay robustness. This integration has significant implications for future organ-on-a-chip development: AI-guided pipelines can enable adaptive experimentation, reduce batch-to-batch variability, and support patient-specific decision frameworks. These insights highlight the emerging role of AI not only as an analytical layer but as a driver of intelligent, self-optimizing microfluidic culture systems.

Despite the rapid adoption of AI in microfluidic and organ-on-a-chip platforms, substantial challenges remain in data analysis and interpretation. These systems routinely generate high-dimensional datasets, including time-lapse microscopy, multiplexed fluorescence imaging, impedance measurements, and biochemical readouts, that require robust pre-processing, segmentation, and feature extraction pipelines before meaningful analysis can occur [74,102]. Variability in chip fabrication, imaging conditions, and biological heterogeneity introduces batch effects that complicate supervised learning and reduce model generalizability. Furthermore, the integration of multimodal data (e.g., combining morphological, electrophysiological, and biochemical features) demands interpretive frameworks capable of linking AI-derived features to underlying biological mechanisms, a task that conventional models struggle to address [78,117]. Dimensionality reduction approaches such as PCA and t-SNE can help visualize phenotypic structure [86,91], but translating these representations into mechanistic or clinically actionable interpretations remains a core bottleneck. Addressing these challenges will require standardized data-processing workflows, biologically informed model architectures, and explainable AI methods that can illuminate the features driving predictions in microfluidic disease models and drug-response assays.

Looking ahead, organ-on-a-chip and microfluidic cell culture systems are expected to evolve into powerful tools for disease modeling, drug discovery, and personalized medicine through the integration of perfusion systems, sensors, and modular multi-organ configurations that collectively simulate a “human-on-a-chip.” Standardized fabrication and modular plug-and-play designs will be necessary for large-scale deployment, while patient-derived cells and 3D bioprinting can enable targeted and personalized applications. When coupled with real-time in situ monitoring and AI-driven analytics, these platforms have the potential to overcome challenges of massive datasets, accelerate mechanistic insights, and enable precision therapeutics. By bridging the gap between preclinical models and clinical translation, the convergence of microfluidics, organ-on-a-chip technology, and explainable AI will pave the way toward more reliable, scalable, and clinically relevant biomedical research models.

Despite these prospects, AI integration in microfluidic and organ-on-chip systems faces several challenges. The lack of feature explainability in many deep learning models limits interpretability and hinders their direct application in biomedical and healthcare contexts, where explainability is critical. This limitation highlights the importance of explainable AI approaches, which can reveal the underlying decision-making process of the models. By incorporating explainable AI, researchers can ensure that predictions regarding cellular behavior, tissue dynamics, or disease modeling remain transparent, interpretable, and clinically relevant. Early efforts toward explainable AI have been demonstrated in nerve-on-a-chip systems [117] and biomedical research [119], and similar adoption is anticipated for microfluidic and organ-on-a-chip applications.

Other technical barriers also constrain the widespread adoption of AI in this domain. High-dimensional datasets, inherent biological variability, data analysis challenges, and experimental noise complicate model training and generalizability. These issues, particularly data analysis problems can be addressed by integrating robust dimensionality reduction methods such as PCA and t-SNE, as well as explainable feature selection strategies such as minimum redundancy maximum relevance (mRMR) [120]. Pre-processing steps, addressing data variability challenges, including multivariate scatter correction [121] and standard normal variate correction [122], can further mitigate noise and inter-sample variation, improving model reliability. Such pre-processing, feature extraction, feature selection, and data analysis techniques combined with superior discriminative capabilities of AI models can significantly reduce the burden of efficient data analysis. Further challenges include generalization and variability problems associated with multi-device systems, which can be addressed using domain adaptation, domain generalization, and efficient data fusion techniques [123,124]. Another practical challenge is the computational demand associated with training and deploying AI models on large-scale experimental data. This requirement may be addressed through lightweight AI architectures optimized for real-time applications and through cloud-based platforms offering scalable computational resources. However, cloud integration raises concerns over data privacy and security, particularly when dealing with sensitive biomedical data. Here, privacy-preserving AI [125] emerges as a promising solution, enabling secure computation without compromising data confidentiality.

In summary, while AI holds significant potential to transform microfluidic cell culture and organ-on-a-chip technologies into intelligent, adaptive, and scalable platforms, several challenges must be addressed. Moving forward, efforts should prioritize the integration of explainable and privacy-preserving AI, the development of lightweight yet high-performance AI models, and closed-loop feedback systems enabling efficient organ-on-a-chip systems in the domain of precision medicine. These advancements will be crucial in exploiting the potential of AI-enhanced microfluidic cell culture and organ-on-a-chip platforms for biomedical research, drug discovery, and personalized medicine.

## 5. Conclusions

Microfluidic cell culture systems and organ-on-a-chip platforms represent a transformative shift in biological process modeling, disease mechanism studies, and drug discovery. The integration of AI, particularly machine learning and deep learning, offers potential in addressing the challenges posed by complex, high-dimensional datasets produced by these platforms. AI can not only enhance data processing and interpretation but also enable closed-loop feedback systems for adaptive experimental control, paving the way for more reliable, physiologically relevant, and reproducible models. However, significant challenges remain, including the lack of fully AI-integrated experimental pipelines, explainability problems with black-box models, and challenges pertaining to standardization, scalability, and data security. Future directions include the adoption of explainable AI, privacy-preserving computational frameworks, and lightweight models optimized for real-time processing. Furthermore, advances in modular organ-chip design, standardized fabrication, and patient-specific models combined with AI-driven analytics are expected to accelerate personalized medicine and translational research. Ultimately, the convergence of microfluidics, organ-on-a-chip systems, and AI will bridge critical gaps between in vitro modeling and clinical applications, establishing these technologies as promising tools for precision health and next-generation biomedical research.

## Figures and Tables

**Table 1:** Overview of machine learning paradigms and their characteristics.

Learning Type	Description	Common Algorithms
**Supervised Learning**	A paradigm where models are trained using labeled datasets, in which each input corresponds to a known output. The objective is to learn a mapping function that generalizes effectively to unseen data, enabling tasks such as classification and regression. While widely applied in predictive modeling, this approach depends on the availability of large, high-quality labeled datasets.	SVM, Random Forest, CNN, ANN, LDA, PLS-DA
**Unsupervised Learning**	Operates on unlabeled datasets to uncover patterns, groupings, or structures within the data. It is frequently applied for clustering, dimensionality reduction, and anomaly detection. Although powerful for exploratory analysis, interpretation of results can be challenging.	k-means, PCA, t-SNE, Autoencoders
**Semi-supervised Learning**	Combines elements of supervised and unsupervised learning by utilizing a small proportion of labeled data together with a larger pool of unlabeled data. The approach improves model performance in scenarios where labeled data are limited, leveraging the intrinsic structure of the unlabeled data.	Self-training, Graph-based methods, Variational Autoencoders
**Reinforcement Learning**	A framework in which an agent interacts with its environment and learns optimal behavior through trial and error, guided by rewards or penalties. It is particularly suited for sequential decision-making and optimization tasks, although it requires well-defined reward functions and often substantial computational resources.	Q-learning, Deep Q-networks (DQN), Policy gradient methods

**Table 2:** Summary of recent studies integrating microfluidic cell culture platforms with AI algorithms for cancer research. The table highlights cancer types, microfluidic device materials and designs, cell types, applied AI approaches, and reported performance metrics.

Cancer Type	Microfluidics	Cells/Organs	AI Algorithm	Performance	Ref.
Cervical cancer	Polyethylene terephthalate (PET) films adhered to 24-well culture slide	H8, HeLa, SiHa	PCA-SVM	Acc = 100%	[81]
Nasopharyngeal carcinoma (NPC)	Three-layered PDMS fabricated using multilayer soft lithography	THP-1, ATCC TIB-202, NP 460, NPC 43	Linear regression	LOD = 5 pg/mL, R^2^ > 0.9	[82]
Multiple cancer types	PDMS using photolithography, graphene oxide quantum dots used to modify microchamber surface	MDA-MB-231, K562, 293T, HEY, SH-SY5Y	k-means, t-SNE	Acc = 95%	[83]
Multiple cancer types	PDMS-based pillar-lattice-array device using standard soft lithography	B16F10, UN-KC6141, CAFs isolated from orthotopic tumors, OT-I CD8^+^ T cells	CNN	AUC = 0.8051	[84]
Pulmonary adenocarcinoma	3D cell culture chip (DAX-1, AIM Biotech)	A549, Peripheral blood mononuclear cells (PBMCs), Primary chicken embryo fibroblasts (CEFs)	Cell-Hunter segmentation algorithm	-	[85]
Cervical cancer, Prostate cancer, Fibrosarcoma	PDMS chip using a combination of 3D stereolithography and soft lithography	HeLa, PC3, HT-1080	PCA, t-SNE, SVM	Sensitivity = 97%, Specificity = 95%, Acc = 96%	[86]
Breast cancer	PDMS rings fixed in culture plates with ZnO nanorods to form cell culture chambers	MDA-MB-231, MCF-7	Unsupervised heirarchical clustering, t-SNE	-	[87]
Breast cancer	PDMS using photolithography	MCF-7, MDA-MB-231, HUVEC	KNN	Acc = 93.8%	[88]
Breast cancer	PDMS, Hydrogel (EKGel)	MCF-7	Gryffin algorithm	-	[89]
Colorectal cancer	PDMS using soft lithography with PEG Hydrogels	Mouse crypts extracted from the small intestines of LGR5–eGFP reporter mice, human colon organoids, human iPSC-derived intestinal organoids, HCT116, human colorectal tissues	LDA, PCA	Pearson’s correlation coefficient PCC = 0.9	[90]
Ovarian cancer	PDMS using soft lithography	Human SKOV3, HEY, A2780, and FTE187 cell lines	PCA, t-SNE, Unsupervised hierarchical clustering	-	[91]
Prostate cancer	PDMS using standard soft lithography	PC-3	YOLOv3, YOLOv5	mAP = 0.97	[92]
Colorectal cancer	Matrigel standard 24-well plate	Patient-derived colorectal organoids	CNN	Acc = 98%	[93]

**Table 3:** Summary of recent applications of AI-integrated microfluidic platforms across diverse domains, highlighting device materials, target cells or organ models, applied AI algorithms, and reported performance metrics.

Application	Microfluidics	Cells/Organs	AI Algorithm	Performance	Ref.
Algae Culture Monitoring	PDMS	*S. quadricauda*	Winograd CNN	Acc = 96.35%	[106]
Cell Cultivation System Analysis	PDMS using soft lithography	*C. glutamicum* ATCC 13032	k-means clustering, Linear regression	-	[107]
High-speed 3D Imaging of Organoid Cultures	PDMS	HCT116, Human embryonic stem cell lines: H1 and K21	StarDist CNN, UNet, YOLOv5, DenseNet121	Acc = 89%	[108]
Detection of Nonbiodegradable Mercury Ions in Gut	PDMS, stainless steel tubes for connecting microchannels	Human colon adenocarcinoma cell line Caco-2	Linear regression	R^2^ = 0.983	[109]
Angiogenesis analysis	PDMS chip, SU8 molds fabricated using conventional photolithography	Human umbilical vein endothelial cells	UNet-like structure with a multimodal bounding box network	-	[110]
Analysis of vascularized microphysiological systems	PDMS using traditional photolithography and soft lithography	INS-1E insulinoma cells, Endothelial cells, Perivascular cells	Deep neural network for regression	R^2^ = 0.88	[111]
Morphological quantification of angiogenic vasculture	96-well polystyrene devices using injection molding bonded with advanced polyolefin diagnostic microfluidic medical tape	Human umbilical endothelial cells, Lung fibroblasts	CNN, Density-based spatial clustering of applications with noise (DBSCAN)	AUC = 0.978	[112]
Bioprocess optimization in dynamic environments	PDMS chip bonded to a glass slide	Yeast strain CEN.PK113-7D	CNN-based StarDist 2D model	-	[113]
Detection of foodborne pathogens	PDMS digital microfluidic chip using photolithography	*S. typhimurium* ATCC 14028, *E. coli* ATCC 43888, *S. aureus* ATCC 25923, *B. cereus* ATCC 14579	TLENTNet based on EfficientNet-B0 CNN	Acc = 97.26%, Sensitivity = 97.25%, Specificity = 97.20%, F1 = 97.19%	[114]
Tissue model evaluation	Chips were manufactured using injection molding and were constructed from cyclic olefin copolymer (COC) with a porous track-etched polyester (PET) membrane	A549, Caco-2, HPMEC, HUVEC, NHBE, HSAEC	MobileNetv3	Acc = 81%, Precision = 0.79, Recall = 0.78	[115]
Probiotic screening	PDMS	Caco-2, HT29-MTX human epithelial cells	PCA	-	[116]
Nerve-on-a-chip, neurological research	PDMS chamber layer and an SU8 2050 photoresist structural layer, gelatin methacryloyl hydrogel layers	Cortical rat neural stem/progenitor cells	PCA, MLP, SVM, SHAP analysis	-	[117]
Evaluation of perfusion cell culture conditions, morphological analysis	Fluid3D-X PET and Fluid3D-X PDMS chips using soft lithography	Caco-2 cells	Convert.ai learning feature of the NIS.ai module in NIS-Elements	-	[118]

## Data Availability

No new data were created or analyzed in this study. Data sharing is not applicable to this article.
